# Treatment outcomes and associated factors among acute coronary syndrome patients admitted to Jimma University Medical Centre, Jimma, Ethiopia: A 5-year retrospective study

**DOI:** 10.1097/MD.0000000000051311

**Published:** 2026-10-30

**Authors:** Elsah Tegene, Gashahun Bekele, Mohammed Mecha, Tamirat Godebo, Edosa Tadasa

**Affiliations:** a Department of Internal Medicine, Institute of Health, Jimma University, Jimma, Ethiopia; b Faculty of Health Sciences, School of Medical Laboratory Sciences, Institute of Health, Jimma University, Jimma, Ethiopia.

**Keywords:** acute coronary syndrome, Ethiopia, in-hospital mortality of ACS, JUMC, treatment outcome of ACS

## Abstract

In developing countries, acute coronary syndrome (ACS) is becoming a more common cause of cardiovascular death. Although thrombolytic treatments and limited percutaneous coronary intervention have been introduced, in-hospital mortality from ACS is still high in sub-Saharan countries. This study aimed to evaluate the treatment outcome of ACS and its associated factors among patients attending Jimma University Medical Center, Jimma, Ethiopia. A retrospective hospital-based cross-sectional study was conducted among 300 ACS patients. Patient records from admissions between November 1st, 2017, and October 31st, 2022 were reviewed, with data collected between December 1^st^ and 31^st^, 2022. Data were collected using a structured checklist, entered, and analyzed using Statistical Package for Social science version 26. Descriptive analysis was used to summarize baseline characteristics and determine treatment outcomes. Bivariable and multivariable regression analyses were employed to determine factors affecting poor treatment outcomes. The odds ratio with a 95% confidence interval was computed, and a *P* value < .05 was considered significant. Among the 300 ACS patients included in our study, 35.3% died during hospitalization. Hypertension was the most common risk factor, present in 88.3% of the patients. In addition, 20.3% were classified as Killip class III or IV. 20.7% had a left ventricular ejection fraction (LVEF) of less than or equal to 30%, and 79.3% had an LVEF > 30%. Regarding treatment, an aspirin loading dose, clopidogrel loading dose, beta-blockers, statins, morphine, and nitrates were given to 284 (94.7%), 286 (95.3%), 227 (75.7%), 283 (94.3%), 109 (36.3%), and 80 (26.7%) patients, respectively. Factors significantly associated with in-hospital mortality were older age (*P* ≤ .001), history of diabetes (*P* = .004), ST-segment elevation myocardial infarction diagnosis (*P* = .003), and Killip class III and IV (*P* ≤ .001), whereas LVEF > 30% was associated with significantly lower odds of in-hospital death (*P* < .001). The proportion of in-hospital mortality attributable to ACS was found to be high. As a result, timely evidence-based therapy should be included in the setup.

## 1. Introduction

Cardiovascular diseases are still the main cause of death worldwide, and the burden is not the same in all regions.^[1]^ Among them, acute coronary syndrome (ACS) encompasses serious and life-threatening conditions caused by reduced blood flow in the coronary arteries, leading to sudden ischemia of the myocardium.^[2]^ ACS includes 3 main conditions: unstable angina (UA), non-ST-segment elevation myocardial infarction (NSTEMI), and ST-segment elevation myocardial infarction (STEMI).^[1,2]^

Despite major progress in the management of ACS,^[3]^ many low- and middle-income countries, especially in sub-Saharan Africa (SSA), are seeing a rapid rise in ACS. This increase is linked to urbanization, unhealthy lifestyle and diet changes, population aging, and poorly controlled cardiovascular risk factors.^[4]^ In high-income countries, the proportion of STEMI among ACS has declined over time.^[1]^ In Ethiopia, published studies have reported a wide range of ACS prevalence, from about 1 to 20%.^[5]^ The growing burden in SSA is especially concerning because many patients are relatively young, and health systems still have limited resources and infrastructure^[4,6]^

Patients with ACS may develop several serious and life-threatening complications, including arrhythmias, heart block, acute heart failure, and cardiac arrest, which can lead to hypoxic-ischemic encephalopathy.^[2]^ Because of this, prompt and aggressive treatment is very important to reduce early complications and improve survival.^[7]^ However, improving the prevention and management of ACS is still difficult in SSA, including Ethiopia.^[8]^ In this region, treatment outcomes are influenced not only by clinical and demographic factors, but also by system-related problems such as delayed arrival after symptom onset, delayed reperfusion therapy, limited numbers of primary emergency facilities, especially in rural areas, and limited prehospital care.^[8]^

In Ethiopia, published studies show that in-hospital mortality among patients with ACS remains high, with reports of 24.5% in northern Ethiopia^[9]^ and 27.4% in central Ethiopia.^[10]^ Another study from central Ethiopia found a recovery rate of 61.8%,^[11]^ showing that outcomes can vary widely across settings. Such varying survival outcomes can be partially attributed to the quality of care and individual-related factors. Nevertheless, evidence on the factors affecting the treatment outcomes among ACS patients in Ethiopia is still limited. Therefore, this study was designed to assess the treatment outcomes of ACS and the associated factors among patients admitted to Jimma University Medical Center (JUMC), Jimma, Ethiopia.

## 2. Methods and materials

### 2.1. Study area, design, and period

The study was conducted at JUMC in Jimma City, which is about 352 km southwest of Addis Ababa, the capital of Ethiopia. It serves as the sole teaching and medical hospital in the southwestern region, providing healthcare services to a large population. The Center had a total bed capacity of 970, of which 220 are dedicated to intensive care units and 750 for inpatients. A retrospective cross-sectional study was conducted. Medical charts of patients who visited the cardiac center from November 1, 2017 to October 31, 2022 were reviewed, and data were collected from December 1 to 31, 2022.

### 2.2. Study populations

All patients aged ≥ 18 years admitted with a diagnosis of ACS who had a registration list in their medical records at the JUMC and who met eligibility criteria.

### 2.3. Eligibility criteria

The study included all complete medical records of patients with ACS above the age of 18 years. Meanwhile, ACS patients with incomplete medical records, who lacked cardiac biomarkers and electrocardiogram, were excluded from this study.

### 2.4. Sample size and sampling technique

The sample size calculation was not applicable because the study used a retrospective cross-sectional design. This study included 300 adults with ACS who had complete data on key variables. A consecutive sampling technique was employed.

### 2.5. Operational definition

Killip class was considered as Class I: no evidence of heart failure; Class II: mild to moderate heart failure, often characterized by S3 heart sound and basal rales (crackling sounds in the lungs); Class III: overt pulmonary edema (fluid buildup in the lungs); and Class IV: cardiogenic shock, a severe condition with hypotension and signs of peripheral vasoconstriction.

### 2.6. Data collection procedure

Data were collected from patients’ medical records using a structured data collection checklist adopted from previously published literature with necessary modifications to fit the local context.^[10,12]^ The data collection format includes question items divided into 3 parts: background information, clinical, and management-related characteristics. Data were gathered from logbook records of the adult patient registry and medical charts over 5 years (from November 1, 2017 to October 31, 2022). The charts of those with a definitive diagnosis of ACS during the specified period were retrieved and reviewed. Two professional healthcare workers were recruited and trained on data collection procedures. This was followed by the introduction and familiarization of the data collectors with the workforce in the record room. Close supervision was maintained during data collection, and checklists were double-checked daily for consistency and completeness by data collectors and the principal investigator before proceeding to data entry and analysis.

### 2.7. Data analysis

Data entry, coding, and cleaning were performed using Epi-info version 7.2.2.2 (Center for Disease Control and Prevention [CDC]) and statistical analysis was done using Statistical Package for Social Science version 26 (IBM Corp.). The baseline characteristics and treatment outcomes of patients were computed using descriptive statistics such as mean ± standard deviation (SD) and median + interquartile range (IQR) for continuous variables, and categorical data were presented as numbers and proportions. Missing values, outliers, and inconsistencies were checked by running frequency distributions and cross-tabulations. Incomplete records with missing key variables were excluded from the final analysis. Binary and multivariable logistic regressions were used to extract factors affecting the outcome variable, poor treatment outcome. Bivariable logistic regression analysis was used to check variables individually associated with the dependent variable. Variables producing *P* values ≤ .25 in the binary regression analysis were then exported into multiple logistic regression models for further analysis to compute the odds ratio with a 95% confidence interval (CI). The Hosmer–Lemeshow Goodness of Fit test was used to check whether the model adequately fit the data in this study and was adequately fitted with a *P* value = .084 > .05. A *P* value was considered significant at the level of < .05. Finally, the study findings were presented using text, tables, and figures.

## 3. Results

### 3.1. Baseline demographic and clinical characteristics of patients

A total of 300 patient medical records were eligible for analysis. The median age of the patients was 60 (IQR, 52–67) years. Males accounted for 216 (72%), and 199 (66.3%) of patients were living in urban areas. Of the ACS patients with complete data, 133 (44.3%) patients presented within 13 to 72 hours of symptom onset, with a mean time from symptom onset to presentation being 79.62 ± 59 hours. The average duration of hospitalization was 8.38 days (SD ± 3.83). The average systolic and diastolic blood pressure of patients during admission was 144.2 (SD ± 45.15) and 85.6 (SD ± 61.33), respectively. Symptom-wise, 272 (90.7%) of the patients had chest pain, and 285 (95%) had shortness of breath. (Table 1).

**Table 1:** Baseline demographic and clinical characteristics of ACS patients admitted to the JUMC, from November 1, 2017 to October 31, 2022.

Variable	Category	Frequency	Percentile (%)	Mean ± SD	Median (IQR)
Age in yrs	< 55	114	38	-	60 (52–67)
	56–64	86	28.7		
	≥ 65	100	33.3		
Sex	Male	216	72	-	-
	Female	84	28		
Residence	Urban	199	66.3	-	-
	Rural	101	33.7		
Time of presentation in hours	≤ 12 hr	47	15.7	79.62 ± 59	-
	13–72 hr	133	44.3		
	> 72 hr	120	40		
SBP (mm Hg)	< 90	23	7.7	144.2 ± 45.15	-
	90–119	31	10.3		
	120–139	54	18.0		
	140–159	89	29.7		
	≥ 160	103	34.3		
DBP (mm Hg)	< 60	23	7.7	85.6 ± 61.33	-
	60-69	31	10.3		
	70–79	54	18.0		
	80–89	89	29.7		
	90–99	103	34.3		
	≥ 100	23	7.7		
Duration of hospitalization (d)	≤ 7	127	42.3	8.38 ± 3.83	-
	8–14	160	53.3		
	≥ 15	13	4.3		
Symptoms	Chest pain	272	90.7	-	-
	Shortness of breath	285	95		
	Nausea and vomiting	67	22.3		
	Cough	173	57.7		
	Sweating	119	39.7		
	Syncope	30	10		

ACS = acute coronary syndrome, DBP = diastolic blood pressure, IQR = interquartile range, JUMC = Jimma University Medical Centre, SBP = systolic blood pressure, SD = standard deviation.

### 3.2. Killip class and risk factors for ACS patients

The majority of patients, 175 (58.3%), had Killip class I during admission. Among the 300 patients, the risk factor profile was as follows: 236 (78.7%) had a history of dyslipidemia, and 265 (88.3%) had a history of hypertension. (Table 2).

**Table 2:** Killip class and risk factors for ACS patients in JUMC, from November 1, 2017 to October 31, 2022.

Variables	Frequency	Percent (%)
Killip class		
I	175	58.3
II	64	21.3
III	48	16.0
IV	13	4.3
Risk Factors		
Dyslipidemia	236	78.7
Hypertension	265	88.3
Diabetes mellitus	158	52.7
Obesity	2	0.7
Family history of heart disease	11	3.7
Previous myocardial infarction	18	6
Exertional angina pectoris	52	17.3
Previous history of Heart failure	31	10.3
Previous stroke	14	4.7
Smoking	98	32.7

ACS = acute coronary syndrome, JUMC = Jimma University Medical Centre.

### 3.3. Class of diagnosis of ACS patients

Regarding the final diagnosis of ACS, the majority of the patients, 202 (67.3%), had STEMI, followed by NSTEMI, 87 (29%), and UA, 11 (3.7%). (Fig. 1).

**Figure 1.**
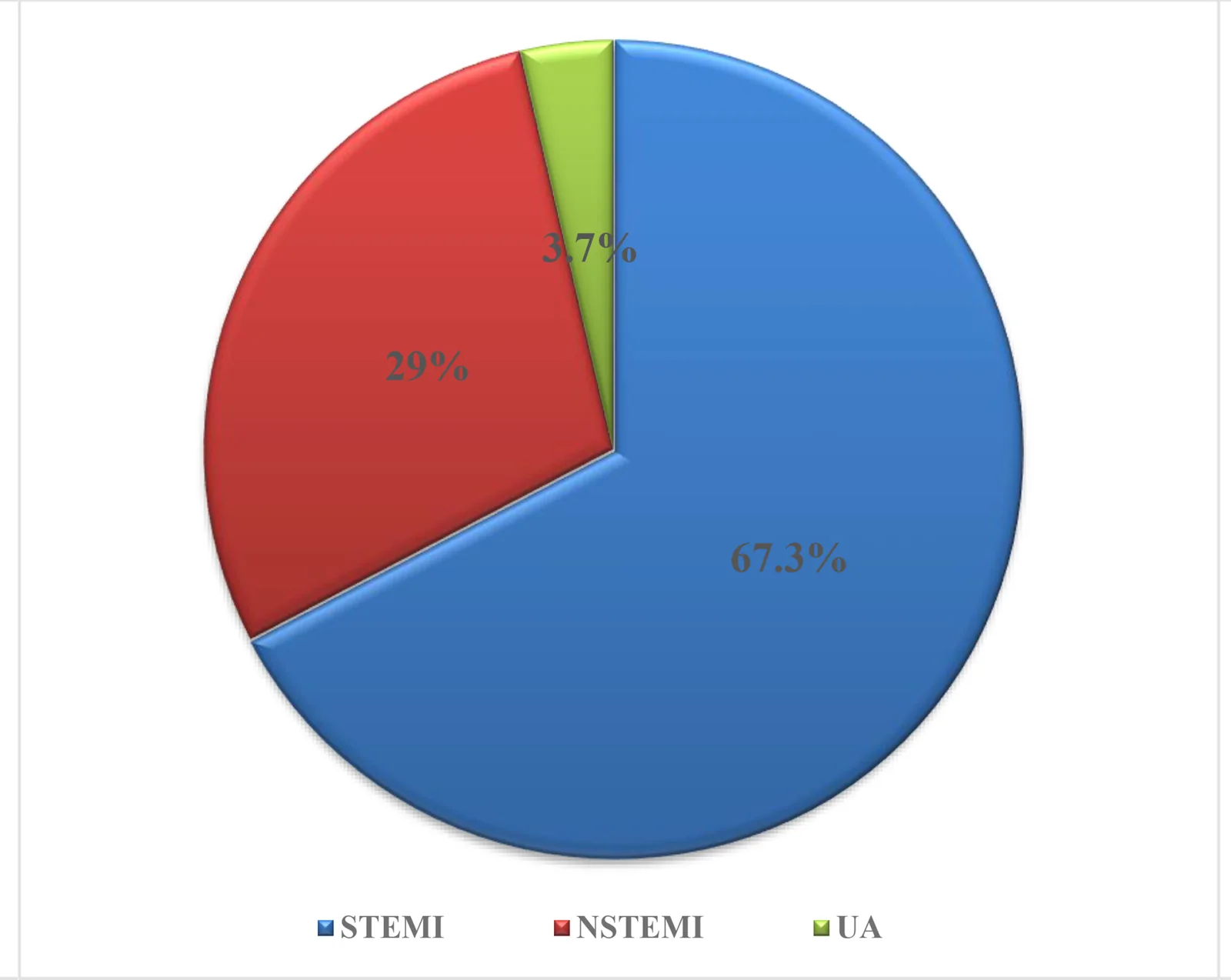
ACS spectrums among patients in JUMC, Jimma, Ethiopia. ACS = acute coronary syndrome, JUMC = Jimma University Medical Center, NSTEMI = non-ST-segment elevation myocardial infarction, STEMI = ST-segment elevation myocardial infarction, UA = unstable angina.

### 3.4. Laboratory and imaging findings

The median (IQR) total cholesterol value was 175.5 (140–246) mg/dL. A high level of total cholesterol (> 200 mg/dL) was recorded in 118 (39.3%) patients, whereas the low density lipoprotein level was > 100 mg/dL in 167 (55.7%) patients. On echocardiographic investigation, 62 (20.7%) had a left ventricular ejection fraction (LVEF) ≤ 30%. Elevated levels of serum creatinine and serum troponin were recorded in 107 (35.7%) and 285 (95%) of them, respectively. (Table 3).

**Table 3:** Laboratory and imaging findings of ACS patients in JUMC, Jimma, Ethiopia, from November 1, 2017 to October 31, 2022.

Variables	Category	Frequency	Percentile (%)	Mean ± SD	Median (IQR)
Total cholesterol	< 200	182	60.7	-	175.5 (140–246)
	≥ 200	118	39.3		
LDL	< 100	133	44.3	-	107 (70–161)
	≥ 100	167	55.7		
HDL	< 40	204	68.0	-	34.5 (30–42)
	≥ 40	96	32.0		
Triglyceride	< 150	158	52.7	-	143 (110–217)
	≥ 150	142	47.3		
LVEF	< 30	62	20.7	-	38 (30–43)
	30–49	202	67.3		
	50–59	29	9.7		
	≥ 60	7	2.3		
SCr	Normal	193	64.3	-	0.98 (0.71–1.4)
	Elevated	107	35.7		
Troponin	Normal	15	5	-	326 (77.25–1506)
	Elevated	285	95		

ACS = acute coronary syndrome, HDL = high density lipoprotein, IQR = interquartile range, JUMC = Jimma University Medical Centre, LDL = low density lipoprotein, SCr = serum creatinine, SD = standard deviation.

### 3.5. Treatment-related factors and in-hospital complications

At presentation and during hospitalization, 284 (94.7%) of patients received a loading dose of aspirin, while 295 (98.3%) continued with a maintenance dose. Similarly, clopidogrel was administered to 286 (95.3%) as a loading dose and to 293 (97.7%) as a maintenance dose. Other commonly administered medications include heparin 283 (94.3%), ACE/angiotensin receptor inhibitor 272 (90.7%), and statin 283 (94.3%). Thrombolysis was administered in 5 (2.5%) of patients with STEMI. Around 194 (64.7%) patients were discharged with improvement. Heart failure was the most prevalent ACS-related complication, affecting 127 patients (42.3%), followed by pulmonary edema in 79 (26.3%). In-hospital mortality was recorded among 106 (35.3%), of which 82 (40.6%) had a diagnosis of STEMI. (Table 4).

**Table 4:** Treatment-related factors and in-hospital complications of ACS patients admitted to JUMC, from November 1, 2017 to October 31, 2022.

Variables	Category	STEMI	NSTEMI	UA	Total
		Frequency N (%)	Frequency N (%)	Frequency N (%)	Frequency N (%)
Medications	Aspirin LD (162–325 mg)	195 (96.5)	79 (90.8)	10 (9.9)	284 (94.7)
	Aspirin MD	200 (99)	84 (96.6)	11 (100)	295 (98.3)
	Clopidogrel LD (300 mg)	195 (96.5)	80 (92)	11 (100)	286 (95.3)
	Clopidogrel MD (75 mg)	198 (98)	84 (96.6)	11 (100)	293 (97.7)
	Heparins	193 (95.5)	83 (95.4)	7 (63.6)	283 (94.3)
	Beta-blocker	163 (80.7)	59 (67.8)	5 (45.5)	227 (75.7)
	ACEI/ARB	187 (92.6)	77 (88.5)	8 (72.7)	272 (90.7)
	Nitrates	41 (20.3)	37 (42.5)	2 (18.2)	80 (26.7)
	Statin	191 (94.6)	83 (95.4)	9 (81.8)	283 (94.3)
	CCB	2 (1)	0	0	2 (0.7)
	Morphine	75 (37.1)	30 (34.5)	4 (36.4)	109 (36.3)
	Pethidine	48 (23.8)	3 (3.4)	0	51 (17)
	Thrombolysis	5 (2.5)	0	0	5 (1.7)
Complications	Heart failure	79 (39.1)	38 (43.7)	10 (90.9)	127 (42.3)
	Pulmonary edema	57 (28.2)	18 (20.7)	4 (36.4)	79 (26.3)
	Major arrhythmia	16 (7.9)	9 (10.3)	0	25 (8.3)
	Stroke	4 (2)	1 (1.1)	0	5 (1.7)
	Cardiogenic shock	18 (8.9)	16 (18.4)	1 (9.1)	35 (11.7)
Outcomes	Death	82 (40.6)	22 (25.3)	2 (18.2)	106 (35.3)
	Discharged	120 (59.4)	65 (74.7)	9 (81.8)	194 (64.7)

ACS = acute coronary syndrome, ARB = angiotensin receptor inhibitor, CCB = calcium channel blocker, JUMC = Jimma University Medical Center, LD = loading dose, MD = maintainance dose, NSTEMI = non-ST-segment elevation myocardial infarction, STEMI = ST-segment elevation myocardial infarction, UA = unstable angina.

### 3.6. Factors associated with in-hospital mortality

Those variables that were found to be significant with a *P* value of < .25 on bivariate logistic regression were exported to multivariate analysis for further association. Age greater than or equal to 65 years (adjusted odd ratio [AOR] = 7.45; 95% CI: 3.27–16.96), history of diabetes mellitus (AOR = 2.64; 95% CI: 1.373–5.09), STEMI diagnosis (AOR = 3.21; 95% CI: 1.483–6.96), and Killip class III & IV (AOR = 10.13; 95% CI: 4.36–23.55) are variables which showed significant association with hospital mortality. Additionally, patients with LVEF > 30% had significantly lower odds of in-hospital mortality than those with LVEF ≤ 30% (AOR = 0.11; 95% CI: 0.051–0.249). (Table 5).

**Table 5:** Logistic regression to determine factors associated with in-hospital mortality in JUMC, Jimma, Ethiopia, from November 1, 2017 to October 31, 2022.

Variable	In Hospital Mortality		COR (95% CI)	*P* value	AOR (95% CI)	*P* value
	Yes (%)	No (%)				
Age						
<55	18 (17)	96 (49.5)	1		1	
56–64	30 (28.3)	56 (28.9)	2.857 (1.461–5.58)	.002	2.328 (0.995–5.447)	.051
≥ 65	58 (54.7)	42 (21.6)	7.365 (3.879–13.98)	< .001	7.448 (3.27–16.96)	< .001
Hypertension						
Yes	99 (93.4)	166 (85.6)	2.386 (1.005–5.66)	.049	2.183 (0.700–6.81)	.178
No	7 (6.6)	28 (14.4)	1.00			
DM						
Yes	71 (67)	87 (44.8)	2.49 (1.523–4.088)	< .001	2.644 (1.373–5.09)	.004
No	35 (33)	107 (55.2)	1		1	
Exertional angina pectoris						
Yes	12 (11.3)	40 (20.6)	0.49 (0.245–0.984)	.045	0.506 (0.196–1.31)	.16
No	94 (88.7)	154 (79.3)	1		1	
Diagnosis						
STEMI	82 (77.4)	120 (61.9)	2.11 (1.229–3.613)	.007	3.213 (1.483–6.96)	.003
NSTEMI + UA	24 (22.6)	74 (38.1)	1.00		1	
Killip class						
I & II	63 (59.4)	176 (90.7)	1		1	
III&IV	43 (40.6)	18 (9.3)	6.674 (3.58–12.42)	< .001	10.132 (4.36–23.55)	< .001
B-blocker						
Yes	68 (64.2)	159 (82)	1.00		1	
No	38 (35.8)	35 (18)	2.54 (1.480–4.356)	.001	2.020 (0.914–4.46)	.082
LVEF						
≤30	47 (44.3)	15 (7.7)	1		1	
>30%	59 (55.7)	179 (92.3)	0.105 (0.055–0.20)	< .001	0.113 (0.051–0.249)	< .001

AOR = adjusted odd ratio, COR = crude odd ratio, CI = confidence interval, JUMC = Jimma University Medical Center, NSTEMI = non-ST-segment elevation myocardial infarction, STEMI = ST-segment elevation myocardial infarction, UA = unstable angina.

## 4. Discussion

As the use of evidence-based therapies and modern interventional procedures improves, the length of hospital stay is expected to decrease. In this study, the average duration of hospitalization was 8.38 days (SD ± 3.83), which is longer than reports from Kenya (5.3 ± 1 day),^[13]^ Brazil (5.1 ± 8.0 days),^[14]^ and the United States (5.56 days).^[13]^ The most common presenting symptoms among the patients were shortness of breath (95%), chest pain (90.7%), and cough (57.7%).

This study found a considerable delay in patients seeking medical care (the average duration of presentation to the hospital was 79.62 ± 59 hours). In our study, 47 (15.7%) of patients were hospitalized within 12 hours, whereas 133 (44.3%) patients presented within 13–72 hours of symptom onset. According to a retrospective study conducted in Kenya, 78% of patients came within 12 hours of symptom onset.^[15]^ Similarly, a study from Dakar, Senegal, found that the average delay before receiving medical care was 14.5 hours.^[16]^ In addition, the European Network for Acute Coronary Treatment study, a pan-European assessment of ACS, found that most patients (65%) arrived within 12 hours of pain onset.^[17]^ The possible reason for significant delay in seeking medical care might be attributed to a lack of knowledge regarding signs of ACS and/or misinterpretation of symptoms by patients.

In the present study, hypertension was the most common risk factor among ACS patients, affecting 88.3% of participants, followed by dyslipidemia in 78.7% and diabetes mellitus in 52.7%. These findings are consistent with a Kenyan study, where hypertension was also the leading risk factor.^[15]^ Similarly, findings from the Saudi Project for Assessment of Coronary Events registry showed a high burden of risk factors, with 58.1% of participants having diabetes, 55.3% hypertension, 41.1% hyperlipidemia, and 32.8% being current smokers.^[18]^ This prevalence of risk factors might be due to inadequate awareness and poor prevention practices among individuals with hypertension, such as limited adherence to lifestyle modification, inconsistent follow-up, and suboptimal compliance with prescribed medications.^[19]^

According to the 2017 European Society of Cardiology 2017 guideline, STEMI patients hospitalized within 12 hours of symptom onset should receive thrombolytic therapy within 30 minutes of first medical contact, provided there is no contraindication. Because STEMI is caused by complete occlusion of a coronary artery, reperfusion should be initiated as early as possible after presentation.^[20]^ In our study, thrombolysis was administered to only 5 (2.5%) of patients with STEMI. This might be related to a time delay between symptom onset and hospital admission, as the mean time to admission was 79.62 ± 59 hours.

In the current study, no patients underwent percutaneous coronary intervention (PCI). PCI is the preferred and most effective reperfusion therapy for symptomatic ACS patients hospitalized within 90 minutes, particularly in severe cases.^[21]^ Although the service has been implemented in the study setting, none of the patients benefited from it. In this study area, PCI was used more often for stable patients than for acutely ill patients. This may be attributed to limited accessibility of PCI service and the high cost of the procedure.

In our study, 94.7% and 95.3% of emergency patients received a loading dose of aspirin and clopidogrel, respectively. This use of dual antiplatelet therapy should be initiated for ACS patients upon admission to the emergency department to prevent cardiovascular complications and mortality.^[20]^ Our findings are also comparable with reports from Greece,^[22]^ where aspirin and clopidogrel were utilized in 97% and 93% of patients, respectively, and from Brazil,^[23]^ where aspirin and clopidogrel were administered to 95% and 88.7% of patients, respectively.

In the present study, the overall in-hospital mortality rate among ACS patients was 35.3%, with STEMI accounting for the largest proportion of deaths (40.6%), followed by NSTEMI (25.3%) and UA (18.2%). These findings indicate that ACS contributes substantially to in-hospital mortality in our setting. One possible explanation is that most patients present with the STEMI form of ACS, which raises the risk of death caused by complete coronary artery occlusion. Similar patterns have been reported in studies from Poland, the Global Registry of Acute Coronary Events registry, and the international registry of ACS in transitional countries^[24–26]^ However, the mortality rate in this study is higher than that reported from Tikur Anbessa Specialized Hospital in Addis Abeba, Ethiopia (27.4%),^[10]^ Mekelle, Ethiopia (24.5%),^[9]^ and Nigeria 21 (.4%).^[27]^ The lower mortality rate reported in those studies may be related to the availability of PCI and thrombolytic therapy in the respective study setting.

This study found a significant association between in-hospital mortality and factors such as old age, a history of diabetes, a diagnosis of STEMI, and a higher Killip class. Importantly, patients with an elevated LVEF of more than 30% had lower odds of death.

Multivariate analysis showed that patients older than 64 years were 7.45 times more likely to die in hospital than those under the age of 55 (AOR = 7.448; 95% CI = 3.27–16.96). This finding aligns with a study undertaken in Ethiopia,^[10]^ which also identified older age as an important predictor of poor ASC outcomes. The possible reason is that as we age, our bodies naturally undergo physiological changes that make them less resilient to the stress of a heart attack, such as decreased cardiac reserve, increased presence of co-morbidities such as diabetes and hypertension, and a higher likelihood of preexisting coronary artery disease, all of which can exacerbate the severity of ACS and impair the body’s ability to recover.

In our study, patients with a history of diabetes were 2.64 times more likely to die from ACS than those without diabetes, which is consistent with a study done in New York^[28]^ showing that diabetes is an independent predictor of poor ACS outcomes. This poorer prognosis may be explained by several interrelated mechanisms, including insulin resistance, hyperglycemia, endothelial dysfunction, enhanced platelet activation and aggregation, more vulnerable plaque characteristics, and a greater extent of coronary lesions, all of which can worsen ischemic injury and reduce survival after ACS.^[29]^

Patients with STEMI were 3.21 times more likely to die in the hospital than those with other types of ACS (AOR = 3.21; 95% CI = 1.483–6.96). Additionally, Killip class III or IV patients were 10.13 times more likely to die in the hospital than Killip class I and II patients. This finding is consistent with a study done in Tikur Anbessa Specialized Hospital, Addis Ababa, Ethiopia^[10]^ where both STEMI diagnosis and higher Killip class were independent predictors of in-hospital mortality. The higher risk associated with STEMI and advanced Killip class likely reflects more extensive myocardial injury, severe hemodynamic compromise, and a greater likelihood of complications like heart failure.

LVEF is the percentage of blood pumped per 1 contraction of the left ventricle.^[30]^ In our study, patients with LVEF > 30% had an 89% lower risk of in-hospital death than those with LVEF < 30% (AOR = 0.113; 95% CI: 0.051–0.249). This may be explained by the fact that in our study, heart failure was present in 35.5% of the fatalities, with advanced Killip class III and IV in 40.6%. The risk of death may be increased in the presence of advanced Killip class heart failure, which results in a lower LVEF.^[31]^ This finding is consistent with a study undertaken at the Ayder Comprehensive Specialized Hospital in Mekelle, Ethiopia,^[9]^ where LVEF <30% was an independent predictor of in-hospital mortality among ACS patients.

This study had several limitations. Because it relied on retrospective medical record data, some relevant variables were unavailable or incompletely documented. In addition, the cross-sectional study design limited our ability to establish causal relationships, and the relatively small sample size may have affected the generalizability of the findings.

## 5. Conclusion

In this study, in-hospital mortality in JUMC was quite high (35.3%). The majority of ACS patients in JUMC (67.3%) were diagnosed with STEMI. Old age, a history of diabetes, a diagnosis of STEMI, and higher Killip class were revealed to be independent predictors of in-hospital mortality in JUMC. Additionally, patients with a raised LVEF of more than 30% were less likely to experience in-hospital mortality. Prospective studies with a greater sample size should be used in future studies for generalization and representation of the population. Evidence-based and timely management should be implemented in the study area.

## Author contributions

**Conceptualization:** Mohammed Mecha.

**Data curation:** Gashahun Bekele, Mohammed Mecha, Tamirat Godebo.

**Formal analysis:** Edosa Tadasa.

**Investigation:** Tamirat Godebo.

**Methodology:** Gashahun Bekele.

**Resources:** Gashahun Bekele.

**Software:** Edosa Tadasa.

**Supervision:** Elsah Tegene.

**Validation:** Elsah Tegene, Tamirat Godebo.

**Visualization:** Mohammed Mecha.

**Writing – review & editing:** Elsah Tegene, Edosa Tadasa.
